# Plasma Proteomics of Diabetic Kidney Disease Among Asians With Younger-Onset Type 2 Diabetes

**DOI:** 10.1210/clinem/dgae266

**Published:** 2024-04-16

**Authors:** Resham Lal Gurung, Huili Zheng, Hiromi Wai Ling Koh, Yiamunaa M, Jian-Jun Liu, Sylvia Liu, Clara Chan, Keven Ang, Clara Si Hua Tan, Radoslaw Mikolaj Sobota, Tavintharan Subramaniam, Chee Fang Sum, Su Chi Lim

**Affiliations:** Clinical Research Unit, Khoo Teck Puat Hospital, Singapore 768828; Cardiovascular and Metabolic Disorders Signature Research Program, Duke-NUS Medical School, Singapore 169857; Clinical Research Unit, Khoo Teck Puat Hospital, Singapore 768828; Institute of Molecular and Cell Biology, Singapore 138673; Clinical Research Unit, Khoo Teck Puat Hospital, Singapore 768828; Clinical Research Unit, Khoo Teck Puat Hospital, Singapore 768828; Clinical Research Unit, Khoo Teck Puat Hospital, Singapore 768828; Clinical Research Unit, Khoo Teck Puat Hospital, Singapore 768828; Clinical Research Unit, Khoo Teck Puat Hospital, Singapore 768828; Clinical Research Unit, Khoo Teck Puat Hospital, Singapore 768828; Institute of Molecular and Cell Biology, Singapore 138673; Clinical Research Unit, Khoo Teck Puat Hospital, Singapore 768828; Clinical Research Unit, Khoo Teck Puat Hospital, Singapore 768828; Clinical Research Unit, Khoo Teck Puat Hospital, Singapore 768828; Institute of Molecular and Cell Biology, Singapore 138673; Diabetes Centre, Admiralty Medical Centre, Singapore 730676; Saw Swee Hock School of Public Heath, Singapore 117549; Lee Kong Chian School of Medicine, Nanyang Technological University, Singapore 308232

**Keywords:** proteomics, younger onset of type 2 diabetes, diabetes kidney disease

## Abstract

**Context:**

Patients with younger onset of type 2 diabetes (YT2D) have increased risk for kidney failure compared to those with late onset. However, the mechanism of diabetic kidney disease (DKD) progression in this high-risk group is poorly understood.

**Objective:**

This work aimed to identify novel biomarkers and potential causal proteins associated with DKD progression in patients with YT2D.

**Methods:**

Among YT2D (T2D onset age <40 years), 144 DKD progressors (cases) were matched for T2D onset age, sex, and ethnicity with 292 nonprogressors (controls) and divided into discovery and validation sets. DKD progression was defined as decline of estimated glomerular filtration rate (eGFR) of 3 mL/min/1.73 m^2^ or greater or 40% decline in eGFR from baseline. A total of 1472 plasma proteins were measured through a multiplex immunoassay that uses a proximity extension assay technology. Multivariable logistic regression was used to identify proteins associated with DKD progression. Mendelian randomization (MR) was used to evaluate causal relationship between plasma proteins and DKD progression.

**Results:**

Forty-two plasma proteins were associated with DKD progression, independent of traditional cardiorenal risk factors, baseline eGFR, and urine albumin-to-creatinine ratio. The proteins identified were related to inflammatory and remodeling biological processes. Our findings suggest angiogenin as one of the top signals (odds ratio = 5.29; 95% CI, 2.39-11.73; *P* = 4.03 × 10^−5^). Furthermore, genetically determined plasma angiogenin level was associated with increased odds of DKD progression.

**Conclusion:**

Large-scale proteomic analysis identified novel proteomic biomarkers for DKD progression in YT2D. Genetic evidence suggest a causal role of plasma angiogenin in DKD progression.

Diabetes is a major cause of kidney failure in many developed countries. Asians, especially those with young onset of type 2 diabetes (YT2D, ie, onset of type 2 diabetes at age <40 years), are a high-risk population for diabetic kidney disease (DKD) (1-3). Possible reasons include longer life-time exposure to diabetes, greater obesity, and glycemic burden (4-6). However, the greater propensity for DKD and accelerated disease progression, reported in several cohorts, suggests a possible pathobiology unique to people with YT2D. Notably, YT2D are underrepresented in most DKD clinical trials. Hence, it is not entirely clear whether the recent advances in DKD treatment can be readily extrapolated to those with YT2D. Therefore, a further deep-dive into understanding the disease mechanism using state-of-the-art molecular epidemiology approach, such as proteomics, among Asians with YT2D will be needed to better inform treatment.

Proteomics offers valuable insights into the pathophysiology of DKD and the identification of novel biomarkers for risk assessment and management. Recent advances in proteomics technology have made considerable inroads to facilitate the simultaneous interrogation of thousands of circulating peptides. For example, Niewczas et al reported that inflammatory proteins such as tumor necrosis factor receptor superfamily 1 (TNFR1) and TNFR2 are associated with the development of end-stage renal disease in type 1 diabetes and T2D (7, 8). However, given the high cost of proteomics assays and the rarity of YT2D relative to those with older onset, there has been a lack of studies dedicated to Asians with YT2D.

To gain deeper insights into disease biology, we collated 436 YT2D DKD progressors and nonprogressors from a few prospective Asian diabetic cohorts with long-term renal follow-up and profiled 1472 targeted proteins using OLINK multiplex proximity extension assays. Next, we evaluated the association of plasma proteins with DKD progression. We also used mendelian randomization (MR) to assess the causal relationship between plasma proteins and DKD progression.

## Material and Methods

### Study Design and Population

This study included YT2D patients from 4 ongoing prospective cohorts (Study of Macro-Angiopathy and micro-vascular reactivity in type 2 diabetes, SMART2D (9); Diabetic Nephropathy, DN (10); Diabetic Kidney Disease-Onset and progression risk factors, DORIS (11); and Maturity-onset diabetes of the Young, MODY (12)) recruited from 2007 to 2019. Detailed descriptions of these cohorts have been described previously. Two broad approaches were used to analyze the data: observational analysis and MR analysis.

**Figure 1.**
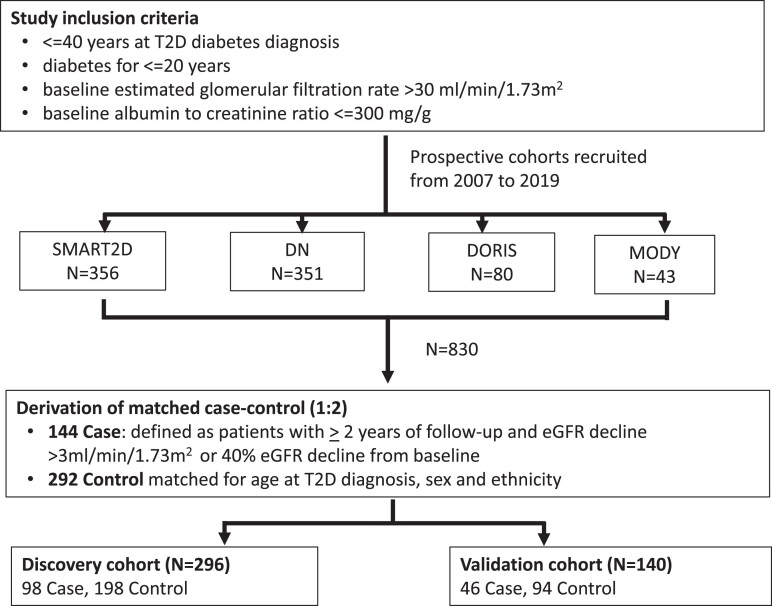
Flowchart of participant selection for observational analysis.

For the observational analysis, we included participants with onset age of T2D at 40 years or younger, diabetes for 20 years or less, baseline estimated glomerular filtration rate (eGFR) greater than 30 mL/min/1.73 m^2^, and baseline urine albumin to creatinine ratio (uACR) less than or equal to 300 mg/g. Among the 830 patients who met the eligibility criteria, 144 participants were DKD progressors (cases). These participants were matched by age at T2D diagnosis, sex, and ethnicity to 292 nonprogressors (controls) in a 1:2 ratio from the pool of eligible participants, resulting in an eventual sample size of 436 participants. Fig. 1 illustrates the study cohorts and participant selection criteria. For the MR analysis, we included multiethnic YT2D participants (T2D onset age ≤40 years) from the SMART2D (9, 13) and DN cohorts (10, 14, 15) with genotype, baseline, and follow-up eGFR readings available. We excluded individuals with baseline eGFR of less than 30 mL/min/1.73 m^2^ to mitigate the confounding effect of chronic kidney disease (CKD) on the relationship between plasma proteins and DKD progression. A total of 867 participants were included (Supplementary Fig. S1 (16)).

The study complied with principles established by the Helsinki Declaration and was approved by the Singapore National Healthcare Group ethical review board. All participants provided written informed consent.

### Clinical and Biochemical Variables

Demographics, baseline clinical data, medical history, and medication usage, were captured by trained research nurses during recruitment. Ethnicity, smoking status, and age at diabetes diagnosis were self-reported. Age at diabetes diagnosis was cross-validated by review of medical records if the reported age was younger than 40 years. Body mass index (BMI) was calculated by weight in kilograms divided by the square of height in meters. Blood pressure was the average of 3 measurements in a sitting position. Glycated hemoglobin A_1c_ (HbA_1c_) was quantified by a point-of-care instrument (DCA Vantage Analyzer, Siemens). High-density lipoprotein cholesterol (HDL), low-density lipoprotein (LDL) cholesterol, serum triglycerides, and creatinine were measured by enzymatic assays (Roche Cobas Integra 700, Roche Diagnostics). Urinary albumin was measured by solid-phase competitive chemiluminescent enzymatic immunoassay (IMMULITE; DPC). Albuminuria was expressed as uACR. Glomerular filtration rate was estimated by CKD–Epidemiology Collaboration equation. Follow-up eGFRs were extracted from electronic medical records captured during routine clinical visits.

### Proteomic Measurements

Baseline plasma samples were analyzed using the Olink platform (Olink Proteomics,), which applies proximity extension assay (17) technology to measure the relative abundance of 1472 proteins from 4 panels: cardiometabolic, inflammation, neurology, and oncology. Cardiometabolic and inflammation panels were selected as cardiovascular disease and inflammation are known to be linked to renal disease pathophysiology based on prior knowledge from experimental and epidemiological studies, while neurology and oncology panels were selected for exploration of novel biomarkers. All samples were processed with quality control conducted by Olink (18). Briefly, 3 interplate controls, 3 negative controls, and 2 sample controls were included in the 96-well plate for interplate and intraplate normalizations and for calculating the limit of detection. Samples were flagged if the internal controls deviated more than 0.3 normalized protein expressions from the plate median. After excluding 18 proteins with >80% samples that failed quality control and 3 proteins that appeared in multiple panels, 1445 unique proteins remained. The intra-assay coefficient of variance ranged between 7% and 8%, while the interassay coefficient of variance ranged between 11% and 14% across the 4 panels.

### Observational Analysis

#### Discovery and validation sets

The matched sets of 144 cases and 292 controls were randomly split into discovery (98 cases/198 controls) and validation (46 cases/94 controls) sets to identify and validate proteins associated with DKD progression. The Wilcoxon rank-sum test was used to compare the continuous variables, and the chi-square test or Fisher exact test was used to compare the categorical variables between cases and controls.

#### Protein-wide analysis

Univariable and multivariable conditional logistic regression models were used to estimate the association between each protein (exposure) and DKD progression (outcome). The multivariable model included baseline clinical characteristics (duration of diabetes, BMI, smoking status, HbA_1c_, systolic blood pressure [SBP], diastolic blood pressure, eGFR, and uACR) as covariates. The models were performed on all proteins assayed in the discovery set, and the Benjamini-Hochberg procedure was used to correct the *P* values for multiple testing. The same analyses were performed in the validation set for proteins that passed the false discovery rate of 10% from the discovery data. A nominal *P* value of less than .05 was used to identify proteins significantly associated with DKD progression in the validation set, and the Benjamini-Hochberg procedure was used to check the *P* values after correcting for multiple testing. Volcano plots were used to visualize the effect size and *P* value of the association between each protein and DKD progression. As the protein expression levels were log2-transformed, the odds ratio (OR) estimated the increase in odds of DKD progression per doubling of protein abundance.

#### Pathway enrichment analysis

Proteins significantly associated with DKD progression in the multivariable model both in the discovery and validation sets were included in the pathway enrichment analyses to elucidate the possible biological mechanisms leading to the decline in kidney function. GeneCodis (version 4) with Gene Ontology (GO) and Kyoto Encyclopedia of Genes and Genomes (KEGG) annotations were used to identify the functions and pathways that involved the differentially expressed proteins.

#### Tissue enrichment analysis

Next, we investigated which tissues the proteins associated with DKD progression were enriched in by using the gene and protein expression data from the Genotype-Tissue Expression (GTEx) (19) and the Human Protein Atlas (HPA) database (20), respectively. Using data from both databases, we mapped the proteins associated with DKD progression to identify tissue-enriched proteins. Specifically, we were interested in kidney, liver, heart, artery, and brain tissues. Furthermore, we looked at a published study that shows the correlation of genes with kidney function (eGFR) or kidney (tubular) fibrosis based on a linear regression model with covariates including age, sex, race, and diabetes and hypertension status (N = 95) (21).

#### Predicting diabetic kidney disease progression using proteins

Among the proteins significantly associated with DKD progression in the multivariable model of both the discovery and validation sets, we used backward stepwise elimination to see which proteins were collectively associated with DKD progression (*P* value <.05 for retention of each protein). Using these proteins, we then evaluated the predictive performance (area under the receiver operating curve, AUC) of adding each of them beyond traditional risk factors for DKD progression in the full cohort.

### Mendelian Randomization Analysis

#### Instrument variable selection for plasma proteins

For proteins that were significantly associated with DKD progression both in the discovery and validation sets, we screened for the availability of their single-nucleotide variations (SNVs, formerly single-nucleotide polymorphisms [SNPs]), the instrumental variables (IVs) for MR analysis, reported in the Asian population from the genome-wide association study (GWAS) catalog data set as of July 10, 2023. For proteins with available IVs, we determined the association between each IV and normalized protein expressions in 2 YT2D subsets from the SMART2D and DN cohorts using linear regression models adjusted for age and sex, stratified by ethnic groups, and meta-analyzed with a fixed-effect model. Heterogeneity in the meta-analyzed data was determined using *I*^2^ statistics, and Cochran's Q *P* value (P_het_) less than .05 was considered significantly heterogeneous. For proteins with more than one genome-wide associated SNV, we selected SNVs that were robustly associated with measured protein in the reported GWAS and in our study population, as well as SNVs that are cis-pQTL.

#### Association of instrumental variable with diabetic kidney disease progression

Logistic regression models were used to determine the association between the selected IVs and DKD progression in the SMART2D and DN cohorts. Model 1 included age and sex. Model 2 included age, sex, BMI, diabetes duration, HbA_1c_, SBP, eGFR, natural log-transformed uACR. Analysis was performed separately in Chinese, Malay, and Indian participants in both cohorts and pooled using fixed-effect meta-analysis. Heterogeneity in meta-analyzed data was determined using *I*^2^ statistics, and P_het_ less than .05 was considered significantly heterogeneous.

#### Mendelian randomization analysis

The validity of MR analysis depends on 3 assumptions: 1) the IV is robustly associated with exposure; 2) the IV must not be associated with confounders, and 3) the IV is only associated with the outcome through the exposure (22). Our approach to select IVs has been described earlier. To assess the validity of the IV, for sensitivity analysis, we investigated the association of IVs selected with cardiorenal traits (HbA_1c_, BMI, SBP, eGFR, and natural log-transformed LDL cholesterol, HDL cholesterol, and uACR) in YT2D cohorts. Furthermore, we searched the GWAS catalog and Phenoscanner (23; July 14, 2023) to identify reported associations of IVs with cardiorenal traits in Asian populations.

Reported genetic proxies from GWAS in Asians was based on adjustment for age, sex, diabetes status, and BMI (24). Hence, for MR analysis, we used estimates of IVs (β _SNV-protein_) from current study populations with protein levels measured. For SNV-DKD (β _SNV-DKD_) coefficients, we included additional 605 (out of 867) YT2D patients with baseline eGFR greater than 30 mL/min/1.73 m^2^, with genotype and follow-up eGFR information available to increase the sample size as described earlier (Supplementary Fig. S1 (16)). We used the Wald ratio to estimate the causal effect (25). All analyses were performed using R version 3.1.2 and Stata SE version 16.0. A 2-sided *P* value of less than .05 was considered statistically significant.

## Results

### Identification of Diabetic Kidney Disease Progression-associated Proteins

**Figure 2.**
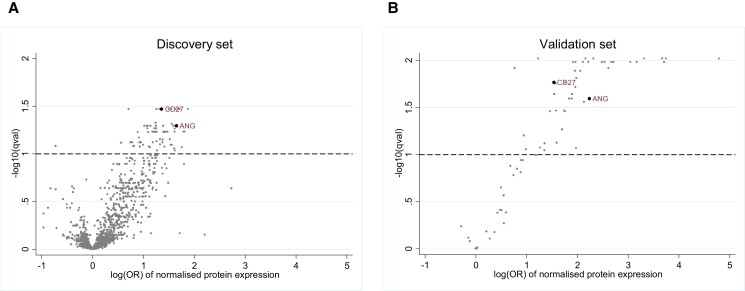
Association between protein and diabetic kidney disease progression among patients in the A, discovery and B, validation sets. The 69 and 42 proteins lying above the dotted line were associated with diabetic kidney disease at 10% false discovery rate in the A, discovery set and 5% nominal type 1 error rate in the B, validation set.

The baseline characteristics between participants in the discovery (N = 296) and validation sets (N = 140) were similar, except that the proportion of Chinese was higher in the validation set (47.9%) than in the discovery set (38.5%) (Table 1). In the discovery set, DKD progressors (cases) were significantly older, had diabetes for a longer duration, poorer glucose control, lower levels of eGFR, and higher levels of uACR than the nonprogressors (controls) (see Table 1). A total of 69 proteins was significantly associated with DKD progression, independent of cardiorenal risk factors, baseline eGFR, and uACR at a 10% false discovery rate (Fig. 2A, Supplementary Table S2 (16)). In the validation set, there were no significant differences in baseline characteristics between cases and controls (see Table 1). Among the 69 proteins identified from the discovery set, 42 proteins were significantly associated with DKD progression in the multivariable adjusted model (nominal *P* < .05) in the validation set (Fig. 2B, Supplementary Table S3 (16)).

**Table 1.** Baseline characteristics of the patients in the discovery and validation sets

Clinical variable	Overall (N = 436)			Discovery (N = 296)			Validation (N = 140)		
	Discovery(N = 296)	Validation (N = 140)	*P*	Control (N = 198)	Case (N = 98)	*P*	Control (N = 94)	Case (N = 46)	*P*
Age at recruitment, y	45 (38-51)	45 (37-50)	.346	**44 (37-50)**	**47 (39-54)**	**.039**	45 (36-50)	45 (37-52)	.427
Age at T2D onset, y	35 (31-38)	34 (28-38)	.186	35 (31-38)	35 (31-38)	.764	34 (27-38)	35 (28-38)	.637
Male	156 (52.7)	66 (47.1)	.278	103 (52.0)	53 (54.1)	.738	45 (47.8)	21 (45.7)	.805
Ethnicity			**.008**			.418			.679
Chinese	**114 (38.5)**	**67 (47.9)**		78 (39.4)	36 (36.7)		45 (47.9)	22 (47.8)	
Malay	**100 (33.8)**	**53 (37.9)**		62 (31.3)	38 (38.8)		34 (36.2)	19 (41.3)	
Indian	**82 (27.7)**	**20 (14.3)**		58 (29.3)	24 (24.5)		15 (16.0)	5 (10.9)	
Body mass index	28.3 (24.2-32.6)	28.9 (24.9-33.3)	.204	28.4 (24.3-33.2)	27.6 (24.1-31.0)	.176	29.0 (24.8-32.5)	28.8 (25.3-36.4)	.682
Smoking status			.579			.661			.849
Never	218 (73.7)	108 (77.1)		149 (75.3)	69 (70.4)		71 (75.5)	37 (80.4)	
Former	17 (5.7)	9 (6.4)		11 (5.6)	6 (6.1)		7 (7.5)	2 (4.4)	
Current	61 (20.6)	23 (16.4)		38 (19.2)	23 (23.5)		16 (17.0)	7 (15.2)	
Diabetes duration, y	10 (5-16)	10 (5-16)	.886	**10 (3-15)**	**13 (8-17)**	**.001**	10 (4-15)	11 (6-18)	.246
HbA_1c_, %	10.8 (8.9-13.0)	10.5 (8.9-12.4)	.201	**10.6 (8.7-12.6)**	**11.9 (9.7-14.3)**	**.001**	10.5 (8.7-11.9)	10.3 (9.2-13.2)	.298
Systolic blood pressure, mm Hg	129 (118-140)	132 (120-145)	.057	128 (117-140)	130 (120-142)	.302	132 (120-142)	131 (118-146)	.831
Diastolic blood pressure, mm Hg	78 (71-86)	80 (73-86)	.269	78 (70-86)	80 (74-86)	.349	80 (72-86)	80 (74-86)	.698
eGFR, mL/min/1.73 m^2^	110.0 (94.4-121.2)	110.1 (98.3-123.2)	.727	**110.9 (96.3-122.0)**	**103.1 (89.8-118.9)**	**.045**	110.1 (101.9-124.4)	109.0 (92.4-119.9)	.168
Follow-up duration, y	5.9 (3.8-8.9)	6.4 (4.3-9.0)	.607	**6.0 (3.8-9.0)**	**5.8 (3.7-8.7)**	**.715**	6.2 (4.2-9.0)	6.9 (4.8-9.0)	.590
No. of follow-up eGFR readings	9 (5-16)	11 (6-16)	.249	**9 (4-14)**	**10 (5-19)**	**.023**	9 (5-14)	13 (8-21)	.002
uACR, mg/g	16 (5-52)	19 (8-58)	.142	**14 (4-40)**	**28 (8-76)**	**.002**	18 (7-46)	25 (12-97)	.078
Total cholesterol, mmol/L	4.47 (3.85-5.15)	4.28 (3.87-5.05)	.557	4.49 (3.94-5.14)	4.26 (3.76-5.16)	.442	4.28 (3.88-5.03)	4.43 (3.83-5.05)	.966
HDL cholesterol, mmol/L	1.14 (0.94-1.36)	1.17 (0.99-1.37)	.616	1.14 (0.96-1.39)	1.14 (0.92-1.34)	.371	1.18 (1.01-1.39)	1.13 (0.99-1.29)	.602
LDL cholesterol, mmol/L	2.74 (2.31-3.45)	2.70 (2.26-3.35)	.650	2.74 (2.34-3.46)	2.74 (2.24-3.41)	.842	2.68 (2.27-3.34)	2.72 (2.22-3.44)	.979
Triglycerides, mmol/L	1.45 (1.02-2.15)	1.53 (1.09-2.04)	.602	1.43 (0.99-2.05)	1.53 (1.03-2.53)	.242	1.51 (1.09-2.19)	1.65 (1.10-2.01)	.593

Data are expressed in median (interquartile range, IQR) for continuous variables and frequency (%) for categorical variables. Variables differed significantly between groups have been highlighted in bold.

Abbreviations: eGFR, estimated glomerular filtration rate; HbA_1c_, glycated hemoglobin A_1c_; HDL, high-density lipoprotein; LDL, low-density lipoprotein; T2D, type 2 diabetes; uACR, urine albumin creatinine ratio.

Combining data from both discovery and validation sets, all 42 proteins were positively associated with DKD progression (Table 2). Of these, at least 7 (CLMP, TNFRSF1A [TNFR1], PTGDS, HAVCR [KIM-1], WFDC2, DSC2, FSTL3) were known biomarkers associated with CKD, albeit in general populations or T2D patients. The strongest associations were observed in CXADR-like membrane protein (CLMP) (OR = 8.66; 95% CI, 3.29-22.80; *P* = 1.26 × 10^−5^), Podocalyxin-like protein (PODXL2) (OR = 7.43; 95% CI, 3.36-16.43; *P* = 7.48 × 10^−7^) and Ephrin type-B receptor 4 (EPHB4) (OR = 7.04; 95% CI, 3.20-15.46; *P* = 1.19 × 10^−6^).

**Table 2.** List of 42 proteins associated with diabetic kidney disease progression in the discovery and validation sets

Uniprot	Gene	Panel	Protein	OR	Lower CI	Upper CI	*P*
P03950	ANG	Cardiometabolic	Angiogenin	5.29	2.39	11.73	4.03E-05
P15291	B4GALT1	Inflammation	β-1,4-galactosyltransferase 1	5.08	2.08	12.39	3.52E-04
P26842	CD27	Oncology	CD27 antigen	3.59	2.05	6.27	7.51E-06
P40259	CD79B	Inflammation	B-cell antigen receptor complex-associated protein β chain	2.85	1.71	4.76	5.96E-05
P22223	CDH3	Neurology	Cadherin-3	3.67	2.11	6.38	4.08E-06
Q07065	CKAP4	Inflammation	Cytoskeleton-associated protein 4	3.30	1.72	6.33	3.16E-04
Q8NC01	CLEC1A	Cardiometabolic	C-type lectin domain family 1 member A	4.57	2.19	9.53	5.12E-05
Q9H6B4	CLMP	Oncology	CXADR-like membrane protein	8.66	3.29	22.80	1.26E-05
P12111	COL6A3	Cardiometabolic	Collagen α-3 (VI) chain	2.81	1.71	4.64	5.03E-05
Q5KU26	COLEC12	Inflammation	Collectin-12	5.96	2.79	12.73	4.10E-06
P78423	CX3CL1	Neurology	Fractalkine	3.95	2.18	7.13	5.41E-06
O00548	DLL1	Oncology	Delta-like protein 1	4.93	2.34	10.38	2.60E-05
Q02487	DSC2	Neurology	Desmocollin-2	4.40	2.27	8.55	1.22E-05
Q9HAV5	EDA2R	Oncology	Tumor necrosis factor receptor superfamily member 27	3.33	2.00	5.54	3.89E-06
P21709	EPHA1	Inflammation	Ephrin type-A receptor 1	3.60	1.84	7.03	1.81E-04
P29317	EPHA2	Oncology	Ephrin type-A receptor 2	4.16	2.09	8.27	4.93E-05
P54760	EPHB4	Cardiometabolic	Ephrin type-B receptor 4	7.04	3.20	15.46	1.19E-06
O95633	FSTL3	Inflammation	Follistatin-related protein 3	3.75	2.02	6.97	2.87E-05
P56159	GFRA1	Oncology	GDNF family receptor alpha-1	4.29	2.32	7.95	3.59E-06
Q96D42	HAVCR1	Oncology	Hepatitis A virus cellular receptor 1	1.91	1.47	2.50	1.56E-06
P22692	IGFBP4	Neurology	Insulin-like growth factor-binding protein 4	2.80	1.74	4.50	2.34E-05
P24592	IGFBP6	Cardiometabolic	Insulin-like growth factor-binding protein 6	5.05	2.46	10.38	1.05E-05
Q6UWL6	KIRREL2	Neurology	Kin of IRRE-like protein 2	3.43	1.85	6.35	9.11E-05
Q86VZ4	LRP11	Cardiometabolic	Low-density lipoprotein receptor-related protein 11	3.95	2.13	7.33	1.37E-05
P36941	LTBR	Inflammation	Tumor necrosis factor receptor superfamily member 3	4.60	2.19	9.67	5.78E-05
Q9H8J5	MANSC1	Oncology	MANSC domain-containing protein 1	5.03	2.44	10.36	1.21E-05
Q96NY8	NECTIN4	Oncology	Nectin-4	4.93	2.43	9.99	9.48E-06
P07196	NEFL	Neurology	Neurofilament light polypeptide	2.05	1.51	2.78	3.57E-06
Q96FE7	PIK3IP1	Neurology	Phosphoinositide-3-kinase-interacting protein 1	6.66	2.94	15.09	5.65E-06
Q9NZ53	PODXL2	Oncology	Podocalyxin-like protein 2	7.43	3.36	16.43	7.48E-07
P41222	PTGDS	Cardiometabolic	Prostaglandin-H2 D-isomerase	5.15	2.52	10.55	7.31E-06
Q969Z4	RELT	Neurology	Tumor necrosis factor receptor superfamily member 19L	5.25	2.38	11.55	3.86E-05
Q6NW40	RGMB	Neurology	RGM domain family member B	4.71	2.11	10.50	1.52E-04
Q9NQ38	SPINK5	Neurology	Serine protease inhibitor Kazal-type 5	3.29	1.79	6.04	1.20E-04
P37173	TGFBR2	Oncology	TGF-β receptor type-2	5.17	2.48	10.78	1.16E-05
Q9GZM7	TINAGL1	Cardiometabolic	Tubulointerstitial nephritis antigen-like	6.17	2.65	14.37	2.40E-05
P19438	TNFRSF1A	Neurology	Tumor necrosis factor receptor superfamily member 1A	4.25	2.20	8.23	1.68E-05
O75509	TNFRSF21	Neurology	Tumor necrosis factor receptor superfamily member 21	4.63	2.22	9.67	4.33E-05
P43489	TNFRSF4	Inflammation	Tumor necrosis factor receptor superfamily member 4	3.69	2.06	6.60	1.14E-05
Q6EMK4	VASN	Cardiometabolic	Vasorin	6.41	2.65	15.49	3.66E-05
Q9Y279	VSIG4	Neurology	V-set and immunoglobulin domain-containing protein 4	3.75	2.06	6.80	1.41E-05
Q14508	WFDC2	Oncology	WAP four-disulfide core domain protein 2	4.15	2.36	7.31	8.35E-07

Data represent effect size of each protein in the combined pool of participants from discovery and validation sets (N = 436).

Adjusted for diabetes duration, body mass index, smoking status, HbA_1c_, systolic blood pressure, diastolic blood pressure, eGFR, and uACR.

Abbreviations: eGFR, estimated glomerular filtration rate; HbA_1c_, glycated hemoglobin A_1c_; OR, odds ratio; uACR, urine albumin creatinine ratio.

### Pathways and Tissue Expression

We conducted pathway enrichment analysis to determine which biological processes and molecular functions in YT2D are related to DKD using the 42 identified proteins. Proteins identified were involved in cell adhesion and microRNA transport (Supplementary Fig. S2 (16)).

Out of the 42 candidate proteins, we found that only WAP four-disulfide core domain 2 (*WFDC2*) gene expression was enriched in kidney tissues but protein expression of folistatin 3 (FSTL3) and insulin growth factor binding protein 6 (IGFBP6) were highly enriched in the kidney tubular cells (Supplementary Fig. S3A (16)). Eight other proteins (VASN, CKAP4, CX3CL1, TNFRSF1A, PTGDS, PODXL2, GFRA1, and HAVCR1) were enriched in either or both the glomeruli and tubules cells in the kidney (Supplementary Fig. S3B (16)). Of the 42 candidate proteins expressing genes, 16 (*ANG*, *CD27*, *CDH3*, *CKAP4*, *COL6A3*, *GFRA1*, *HAVCR1*, *IGFBP4*, *IGFBP6*, *LTBR*, *NEFL*, *TINAGL1*, *TNFRSF21*, *TNFRSF4*, *VSIG4*, *WFDC2*) genes were significantly correlated with CKD and kidney fibrosis in general populations (Supplementary Table S4 (16)) (21).

### Prognostic Utility of Proteins

We examined the prognostic utility of the 42 identified proteins for predicting future DKD progression (Supplementary Fig. S4 (16)). Traditional risk factors (age, sex, ethnicity, BMI, smoking status, diabetes duration, HbA_1c_, SBP, eGFR, and uACR) collectively predicted DKD progression with an AUC of 0.71 (95% CI, 0.65-0.76). Among the 42 proteins identified, backward stepwise elimination retained PODXL2, KIRREL2, CX3CL1, and HAVCR1 in the model, which collectively had an AUC of 0.76 (95% CI, 0.71-0.82). Adding these 4 proteins to the demographic/cardiorenal risk factors increased the predictive AUC to 0.79 (95% CI, 0.73-0.84), an improvement of 0.08 (*P* < .001)

### Association of Instrument Variable With Plasma Proteins

IVs were available only for 2 plasma proteins in Asians (26) (Supplementary Table S5 (16)). The risk variant rs1042445 (*P* = 2 × 10^−28^) and rs2228243 (*P* = 2 × 10^−45^) were associated with plasma CD27 antigen while rs17114699 (ANG) (*P* = 7 × 10^−30^) and rs11624221 (OR6S1) (*P* = 9 × 10^−14^) were associated with plasma angiogenin. The risk variants rs1042445 and rs2228243, and rs17114699 and rs11624221 were in linkage disequilibrium. Therefore, rs1042445 and rs11624221 were excluded.

The risk variant rs17114699 [T] was significantly associated with plasma angiogenin level in YT2D in all 3 ethnic groups, with the strongest association observed among Chinese (meta-β = 0.262, SE = 0.052; *P* = 4.61 × 10^−7^; *P*_het_ = 0.438) adjusted for age and sex (Supplementary Table S6 (16)). The risk variant rs2228243 [G] was not associated with plasma CD27 antigen in all ethnic groups. Similar results were observed when meta-analyzed.

### Association Between Instrumental Variable and Diabetic Kidney Disease Progression

Among the 867 YT2D participants from the SMART2D and DN cohorts, 321 (37%) experienced DKD progression (see Supplementary Fig. S1 (16)). The rs17114699 [T] allele was associated with increased odds of DKD progression (meta-OR = 1.49; 95% CI, 1.08-2.08; *P* = .018; *P*_het_ = .578) after adjustment for age and sex (Table 3), which remained significant in the multivariable adjusted model (meta-OR = 1.51; 95% CI, 1.01-2.26; *P* = .043; *P*_het_ = .747). Among the 3 ethnic groups, the association of the rs17114699 [T] allele with DKD progression was directionally consistent but a significant association with DKD progression was observed only in Chinese individuals (meta-OR = 1.74; 95% CI, 1.05-2.87; *P* = .032; *P*_het_ = .675).

**Table 3.** Association of rs17114699 with diabetic kidney disease progression

		Meta (N = 867)					SMART2D (N = 539)			DN (N = 328)		
	Group	Control/Case	OR (95% CI)	*P*	*P*_het_	*I* ^2^	Control/Case	OR (95% CI)	*P*	Control/Case	OR (95% CI)	*P*
**Model 1**	**All**	**546/321**	**1.49 (1.08-2.08)**	**.018**	**.578**	**0.0**						
	Chinese	308/144	1.78 (1.19-2.69)	.006	.320	0.0	200/75	1.54 (0.94-2.54)	.088	108/69	2.40 (1.18-4.92)	.016
	Malay	97/106	1.12 (0.54-2.31)	.763	.647	0.0	59/53	0.99 (0.40-2.44)	.975	38/53	1.40 (0.42-4.71)	.585
	Indian	141/71	1.00 (0.41-2.42)	.991	.494	0.0	110/42	0.81 (0.28-2.35)	.698	31/29	1.59 (0.32-7.92)	.573
**Model 2**	**All**	**533/300**	**1.51 (1.01-2.26)**	**.043**	**.747**	**0.0**						
	Chinese	300/131	1.74 (1.05-2.87)	.032	.675	0.0	200/73	1.61 (0.86-2.99)	.134	100/58	2.02 (0.85-4.79)	.112
	Malay	94/102	1.33 (0.54-3.26)	.534	.235	29.1	58/53	0.93 (0.32-2.72)	.890	36/49	3.01 (0.60-15.21)	.182
	Indian	139/67	1.05 (0.39-2.82)	.930	.649	0.0	109/41	0.91 (0.28-2.92)	.867	30/26	1.51 (0.23-9.86)	.665

OR was calculated per increase in risk allele (T) for elevated plasma angiogenin levels after adjustments.

Model 1: adjusted for age, sex. Model 2: adjusted for age, sex, BMI, eGFR, diabetes duration, HbA_1c_, SBP, eGFR, and ln uACR.

Abbreviations: BMI, body mass index; DN, Diabetic Nephropathy; eGFR, estimated glomerular filtration rate; HbA_1c_, glycated hemoglobin A_1c_; OR, odds ratio; *P_het_*, Cochran *Q*-test heterogeneity; SBP, systolic blood pressure; uACR, urine albumin creatinine ratio.

### Mendelian Randomization

MR analyses were performed for plasma angiogenin due to the availability of a reliable instrument variable. The effect size of genetic instruments identified from GWAS in Asians (26) was derived from the meta-analysis in the present study. Similarly, meta-analyzed data on the association of rs17114699 with DKD progression in our present study were used for MR analysis. Genetic predisposition for higher level of plasma ANG was associated with an increased OR of 4.03 (95% CI, 1.28-12.68; *P* = .017) for DKD progression per unit increase in plasma ANG (Table 4).

**Table 4.** Mendelian randomization analysis on association of plasma angiogenin with diabetic kidney disease progression in younger onset of type 2 diabetes

Instrumental variable	EA	OA	EAF*^a^*	β (SE) of plasma ANG per allele	*P*	OR (95% CI) for DKD per allele	*P*	MR estimate for DKD per 1-unit increase in plasma ANG	*P*
rs17114699	T	G	0.11	0.286 (0.041)	3.72 × 10^−12^	1.49 (1.08-2.08)	.018	4.03 (1.28-12.68)	.017

Data are adjusted for age and sex.

Abbreviations: DKD, diabetic kidney disease; EA, effect allele; EAF, effect allele frequency; MR, mendelian randomization; OA, other allele; OR, odds ratio.

^*a*^EA in the study population. OR was calculated per doubling (1 unit) of plasma angiogenin after adjustment for age and sex.

In sensitivity analysis, the rs17114699 risk variant was not associated with clinical risk factors for cardiorenal complications (HbA_1c_, BMI, SBP, eGFR, natural log-transformed HDL, LDL, and uACR) in the YT2D population (Supplementary Table S7 (16)). Additionally, there was no reported significant association of rs17114699 with other clinical traits in East Asians and South Asian populations in the Phenoscanner (23; July 14, 2023).

## Discussion

In this prospective study of Asians with YT2D, we performed a comprehensive analysis of circulating proteins to facilitate the identification of markers of DKD progression, independent of traditional cardiorenal risk factors, including baseline kidney function, and to gain insight into relevant biological pathways. The present study uses data from several cohorts to identify 42 proteins associated with DKD proregression. The proteins identified were mainly related to inflammatory and cell adhesion pathways. In addition, MR analysis indicated a causal association of plasma ANG with DKD progression. This discovery work provides directions for future mechanistic and clinical studies to deepen the understanding of the molecular etiology of DKD as well as facilitate drug development for DKD.

The present study is the first to describe the association of several plasma proteins with DKD progression in YT2D. All identified proteins had a positive association with DKD progression, with higher levels associated with deteriorating kidney function. Notably, the increased plasma proteins levels preceded the development of CKD as the median eGFR of the study population was 110 mL/min/1.73 m^2^. Among the proteins identified, some were consistent with previous reports in T2D (TNFRSF1A (27), KIM-1 (28), PTGDS (28)) or in general populations (LMP, DSC2, FSTL3 and WFDC2 (29, 30)). Importantly, to our knowledge, the association of several proteins identified with DKD progression in YT2D were novel.

The strongest association with DKD progression was observed with increased plasma levels of CXADR-like membrane protein (CLMP), podocalyxin-like protein 2 (PODXL2) and Ephrin type-B receptor 4 (EPHB4). CLMP is a type 1 transmembrane protein, localizses to junctional complexes, and is suggested to have a role in cell-cell adhesion. Several studies have shown its importance in adipocyte maturation (31), intestinal development (32), and heart diseases (33). Interestingly, PODXL2, also named endoglycan, is a type I transmembrane glycoprotein and a CD34 family member with homology to podocalyxin (34), and data from animal studies suggest it may be important in lumen formation in the nephron (35). EPHB4 is a tyrosine kinase receptor and, together with its ligand, may be involved in blood vascular morphogenesis (36). Consistent with data from clinical and animal models showing increased expression of WFDC2 in kidney diseases, we found that elevated plasma WFDC2 is associated with DKD progression. WFDC2 is a molecular marker of tubulointerstitial fibrosis and tubular cell damage in patients with CKD and is mainly expressed in tubular cells (37, 38). Our findings suggest increased plasma WFDC2 as an early marker for DKD progression in the YT2D population.

Our findings from MR analysis provide the first evidence of the prospective association of plasma ANG and DKD progression. Angiogenin, a secreted ribonuclease, is highly expressed in the liver, and previous work has implicated this protein in the pathophysiology of cancer, neurological disorders, and cardiovascular diseases (39, 40). A recent cross-sectional study including 108 participants showed that plasma ANG levels also increased with advanced CKD (41). Although gene expression of ANG in kidney tissues decreased with eGFR decline and fibrosis in the general population, our observational and MR analyses in YT2D suggest that elevated plasma ANG increases the risk of DKD progression. Given its importance in angiogenesis, corroboration between our observation and previous findings highlights the importance of angiogenesis for DKD progression.

The strength of this study is worth noting. To our knowledge, this is a comprehensive proteome analysis evaluating the association of plasma proteins with DKD progression in Asian YT2D patients. The clinical outcome was based on eGFR with long-term follow-up. In addition, to ensure validity of our MR, i) we used IVs robustly associated with plasma angiogenin reported in Asian populations and validated in YT2D participants; and ii) a cis-pQTL as IVs because they are considered to have a more direct and specific biological effect on the proteins compared to trans-pQTLs (42); and are not associated with known cardiorenal risk factors linked with DKD progression. Based on a literature search, there was also a lack of evidence for the pleiotropic effect. However, several limitations should be highlighted in the present study. First, our findings are based on Asian individuals, and whether our findings can be generalized to other populations needs further investigation. Second, although we used all 4 Olink panels, we cannot consequently ascertain that we did not miss essential proteins not on the list. In addition, the proteomics assay does not provide standard concentration units, making comparisons with clinically applied cutoffs difficult. However, we observed that proteins measured by the Olink platform and by conventional enzyme-linked immunosorbent assay are highly correlated for most proteins (Supplementary Table S8 (16)). Third, due to a lack of independent replication studies, we split our Y2TD cohorts into discovery and validation sets, resulting in a smaller sample size. Fourth, IVs were unavailable for most of the proteins identified; hence, their causal relationship with DKD progression could not be evaluated. Finally, there were some overlaps of samples for estimating the IV exposure and IV outcome (262/867) for MR analysis. However, the IVs for plasma ANG was not associated with selected well-known cardiorenal risk factors, and sensitivity analysis suggested a lack of pleiotropic effects. Nevertheless, we cannot entirely exclude the possibility of pleotropic effects. The MR estimates, as observed in our study, were also larger than those from conventional observational analysis, but this could be attributed to long-term effects of genetic variants on the exposure (plasma ANG level).

In conclusion, the large-scale proteomic analysis identified 42 known and novel proteins associated with DKD progression in the YT2D population. We were able to refine biological mechanisms further and focus on a limited number of biological processes, namely inflammation and remodeling. Furthermore, we provide evidence of a potential causal role of plasma ANG in promoting DKD progression in YT2D. Notably, this study is exploratory, with findings that need to be verified in other larger cohort studies.

## Data Availability

Restrictions apply to the availability of the data generated or analyzed during this study to preserve patient confidentiality or because they were used under license. The corresponding author will on request detail the restrictions and any conditions under which access to some data may be provided.

## Abbreviations

AUC: area under the receiver operating curve
BMI: body mass index
CKD: chronic kidney disease
DKD: diabetic kidney disease
DN: diabetic nephropathy
eGFR: estimated glomerular filtration rate
GWAS: genome-wide association study
HbA_1c_: glycated hemoglobin A_1c_
HDL: high-density lipoprotein
IV: instrumental variable
LDL: low-density lipoprotein
MR: mendelian randomization
OR: odds ratio
P_het_: Cochran's Q *P* value
SBP: systolic blood pressure
SMART2D: Study of Macro-Angiopathy and micro-vascular reactivity in type 2 diabetes
SNV: single-nucleotide variation
uACR: urine albumin-to-creatinine ratio
YT2D: younger onset of type 2 diabetes
